# *Punica granatum* L. Leaf Extract Attenuates Lung Inflammation in Mice with Acute Lung Injury

**DOI:** 10.1155/2018/6879183

**Published:** 2018-02-20

**Authors:** Aruanã Joaquim Matheus Costa Rodrigues Pinheiro, Jaciara Sá Gonçalves, Ádylla Wilenna Alves Dourado, Eduardo Martins de Sousa, Natilene Mesquita Brito, Lanna Karinny Silva, Marisa Cristina Aranha Batista, Joicy Cortez de Sá, Cinara Regina Aragão Vieira Monteiro, Elizabeth Soares Fernandes, Valério Monteiro-Neto, Lee Ann Campbell, Patrícia Maria Wiziack Zago, Lidio Gonçalves Lima-Neto

**Affiliations:** ^1^Programa de Pós-Graduação, Universidade CEUMA, São Luís, MA, Brazil; ^2^Programa de Pós-Graduação da Rede BIONORTE, Universidade Federal do Amazônas, Manaus, AM, Brazil; ^3^Departamento de Farmácia, Faculdade Pitágoras, São Luis, MA, Brazil; ^4^Laboratório de Estudos Ambientais, Instituto Federal do Maranhão, São Luís, MA, Brazil; ^5^Laboratório de Farmacognosia I, Universidade Federal do Maranhão, São Luís, MA, Brazil; ^6^Departamento de Medicina, Universidade CEUMA, São Luís, MA, Brazil; ^7^Centro de Ciências Biológicas e da Saúde, Universidade Federal do Maranhão, São Luís, MA, Brazil; ^8^Department of Global Health, University of Washington, Seattle, WA, USA; ^9^Departamento de Odontologia, Universidade CEUMA, São Luís, MA, Brazil

## Abstract

The hydroalcoholic extract of *Punica granatum* (pomegranate) leaves was previously demonstrated to be anti-inflammatory in a rat model of lipopolysaccharide- (LPS-) induced acute peritonitis. Here, we investigated the anti-inflammatory effects of the ethyl acetate fraction obtained from the pomegranate leaf hydroalcoholic extract (EAFPg) on the LPS-induced acute lung injury (ALI) mouse model. Male Swiss mice received either EAFPg at different doses or dexamethasone (per os) prior to LPS intranasal instillation. Vehicle-treated mice were used as controls. Animals were culled at 4 h after LPS challenge, and the bronchoalveolar lavage fluid (BALF) and lung samples were collected for analysis. EAFPg and kaempferol effects on NO and cytokine production by LPS-stimulated RAW 264.7 macrophages were also investigated. Pretreatment with EAFPg (100–300 mg/kg) markedly reduced cell accumulation (specially neutrophils) and collagen deposition in the lungs of ALI mice. The same animals presented with reduced lung and BALF TNF-*α* and IL-1*β* expression in comparison with vehicle controls (*p* < 0.05). Additionally, incubation with either EAFPg or kaempferol (100 *μ*g/ml) reduced NO production and cytokine gene expression in cultured LPS-treated RAW 264.7 macrophages. Overall, these results demonstrate that the prophylactic treatment with EAFPg attenuates acute lung inflammation. We suggest this fraction may be useful in treating ALI.

## 1. Introduction

Acute lung injury (ALI) is a clinical condition that causes disruption of the lung endothelial tissue and epithelial barrier and loss of lung function [1, 2]. ALI incidence remains high, and it is associated with high rates of mortality and morbidity worldwide, especially in developing countries [3]. ALI is characterized by intense transepithelial leukocyte infiltration, exudate accumulation in the lungs, loss of integrity of the alveolar-capillary membrane, and tissue damage [4]. This response has been suggested to be due to the increased production of inflammatory mediators including cytokines (TNF-*α*, IL-1*β*, and IL-6), especially by alveolar macrophages and neutrophils [5].

*Punica granatum* L. (pomegranate) has been shown to possess wound healing, antimicrobial, antioxidant, and anti-inflammatory properties [6–8]. Especially due to its anti-inflammatory activities, pomegranate is traditionally used to treat infections. In this context, we recently demonstrated that the leaf hydroalcoholic extract obtained from pomegranate is anti-inflammatory as it inhibits TNF-*α* production and decreases neutrophil migration in a rat model of lipopolysaccharide- (LPS-) induced acute peritonitis [8]. Furthermore, Haseeb et al. showed that the pomegranate fruit extract attenuates IL-6 production, reactive oxygen species, IL-1*β*-mediated phosphorylation of the inhibitor of nuclear factor kappa-B kinase subunit beta (IKK*β*), expression of IKK*β* mRNA, degradation of I*κ*B*α*, and the activation and nuclear translocation of NF-*κ*B/p65 in human chondrocytes [9].

To date, pomegranate effects on acute lung inflammation have not been yet investigated. Here, we assessed the anti-inflammatory potential of the ethyl acetate fraction (EAFPg) obtained from a pomegranate leaf hydroalcoholic extract in a mouse model of LPS-induced acute lung injury.

## 2. Material and Methods

### 2.1. Plant Material and Preparation of EAFPg

Fresh leaves of *Punica granatum* L. were collected at the Ático Seabra Herbarium of the Universidade Federal do Maranhão in Sao Luis, Maranhão, Brazil [8], and a voucher specimen was deposited (voucher number 01002). The leaf hydroalcoholic extract was prepared, and the EAFPg was obtained as previously described [8].

### 2.2. HPLC-DAD-ESI-IT/MS Analysis

The chemical constituents of EAFPg were analyzed in a high-performance liquid chromatography (HPLC) system (LC-10AD, Shimadzu) equipped with a photodiode array detector coupled to an Esquire 3000 Plus ion trap mass spectrometer (Bruker Daltonics, Bremen, Germany), using electrospray ionization (ESI) as previously described [10]. Separation was performed using a Phenomenex Kinetex C-18 column (250 × 4.6 mm, 5 *μ*m; Torrance, CA, USA). The column oven was maintained at room temperature. The HPLC was set up with an elution gradient as follows: 0–2 min, 5% B; 2–10 min, 5–25% B; 10–20 min, 25–40% B; 20–30 min, 40–50% B; 30–40 min, 50–60% B; and 40–50 min, 70–100% B. Acetic acid (2%) in Milli-Q water was used as mobile phase A, and methanol was used as mobile phase B. The injection volume consisted of 25 *μ*l of the reconstituted sample at 5 mg/ml, with a flow rate of 0.6 ml/min. Detection was achieved in a diode array detector (DAD) at 200–500 nm and with direct mass spectrometry/mass spectrometry, a method in negative electrospray (-ESI) mode with a detector voltage maintained at 4.0 kV, ion source at 40 V, and capillary temperature at 320°C. The nebulizing gas was nitrogen (N_2_) flowing at 7 ml/min, a sheath gas provided at a pressure of 27 psi, while helium was used as the collision gas. Analyses were carried out using full-scan mass spectra and data-dependent MS^2^ scans from *m*/*z* 100 to 2000. The compounds were identified on the basis of their molecular ion mass fragmentation. The obtained mass spectrum was compared with that of the literature.

### 2.3. In Vivo Assays

#### 2.3.1. Animals

Sixty-six nonfasted male outbred Swiss mice (20–30 g) were used. Mice were obtained from the animal facility of Universidade CEUMA (UNICEUMA) and were kept in a climatically controlled environment (room temperature of 22 ± 2°C and humidity of around 60%) under 12 : 12 h light-dark cycle (lights on 07:00 a.m.). All procedures were approved by the Ethics Committee of UNICEUMA (April 24, 2014; #107/2014) and carried out in accordance with the Brazilian Society for Animal Welfare (SBCAL).

#### 2.3.2. Induction of LPS-Induced ALI and Pharmacological Treatments

Animals were randomly distributed into six groups (*n* = 6/group) for lung analysis and five groups (*n* = 6/group) for bronchoalveolar lavage fluid (BALF) analysis. Mice received a single oral administration (p.o.) of vehicle (saline, 10 ml/kg), EAFPg (30–300 mg/kg), or dexamethasone (5 mg/kg) 30 min prior to LPS challenge. ALI was induced by a single intranasal (i.n.) instillation of LPS (30,000 EU in 50 *μ*l/animal and 25 *μ*l/narine). Vehicle-treated mice were used as controls. Four hours after the challenge, mice were culled by anesthetic overdose with ketamine (120 mg/kg, i.p.) and xylazine (10 mg/kg, i.p.), and the bronchoalveolar lavage fluid (BALF) was collected. For this, the trachea of each mouse was cannulated and the alveoli were washed three times with 0.5 ml of ice-cold phosphate buffered saline (PBS) containing 0.5% sodium citrate. An aliquot of the BALF was separated and used for quantification of leukocyte counts and the rest centrifuged at 400 ×g for 10 min at 4°C. The supernatant was immediately frozen and stored at −80°C for further analysis. In a separate series of experiments, the lungs were collected from animals that did not undergo BALF collection and were processed for histological analysis.

#### 2.3.3. Total and Differential Leukocyte Counts in BALF Samples

Total cell counts were performed in a Neubauer chamber and microscope (Zeiss Axio Imager Z2 upright microscope, Carl Zeiss, Göttingen, Germany) after diluting an aliquot of the BALF with Türk solution (1 : 20). Another aliquot (50 *μ*l) was used for differential cell counts. For this, the slides were stained with May-Grünwald Giemsa and then analyzed by microscopy in an immersion objective. A minimum of 200 leukocytes was considered. Two independent researchers performed blinded analyses; if there was discordance, a third researcher performed the analysis.

#### 2.3.4. RT-qPCR Assays in the Lung Tissue

TNF-*α*, IL-6, IL1-*β*, and IL-10 mRNA expression in lung samples was determined by RT-qPCR. Total RNA was extracted by using a RNAeasy Mini Kit (Qiagen, Hilden, Germany) according to the manufacturer's instructions in a QIAcube automatic DNA extractor (Qiagen, Hilden, Germany). Samples were treated with DNase (Qiagen, Hilden, Germany) and then reverse-transcribed using 200 U of SuperScript II reverse transcriptase (Thermo Fisher Scientific) to obtain cDNA. The qPCR assays were carried out in 96-well plates using a double-stranded DNA dye (GoTaq® real-time PCR Master Mix from Promega) in a QuantStudio™ 6 flex real-time PCR instrument as previously described. GAPDH mRNA was used as an endogenous reference gene. mRNA relative expression was calculated using the 2^−ΔCt^ method [11]. Results represent the mean values obtained from two independent experiments, with assays performed in triplicate.

#### 2.3.5. Cytokine Levels in BALF Samples

BALF TNF-*α* and IL-10 protein levels were analyzed in enzyme-linked immunosorbent assay kits according to the manufacturer's instructions (R&D Systems). Sample readings for each cytokine were compared with those of a standard curve (0–800 pg/ml). Results are expressed as protein levels in pg/ml. All experiments were performed in triplicate.

#### 2.3.6. Albumin Levels in BALF Samples

Albumin levels in BALF samples were measured by the Bradford method using the standard Quick Start Bradford Protein Assay Kit from Bio-Rad (Hercules, California) according to the manufacturer‘s instructions. Albumin was measured as it is an indicator of plasma extravasation. Absorbance was measured in an automated spectrophotometer at 650 nm. Readings obtained were compared with those of a standard curve of albumin (0–2.0 mg/ml). Results are expressed as mg of albumin per ml of BALF. All experiments were performed in triplicate.

#### 2.3.7. Histological Analysis

The collected lungs were fixed in 10% neutral formalin for 24 h and then embedded in paraffin. After deparaffinization and dehydration as previously described [10], sections (4 *μ*m) were stained with hematoxylin and eosin or Masson's trichrome and analyzed by microscopy under 100x and 400x magnification. The density of leukocytes/unit area (130 *μ*m^2^) was determined by counting the number of cells on the integrating eyepiece that fell into areas of the bronchioli and distal lung parenchyma using the ImageJ Program. Using the same program, the collagen area was measured also in areas of the bronchioli and distal lung parenchyma. All measurements were performed in ten random sections of 130 *μ*m^2^ per animal at 200x (for collagen area) and 400x magnification (for leukocyte count), and the researcher who performed the analysis was unaware of the experimental group designation. Two independent researchers performed blinded analyses; if there was discordance, a third researcher performed the analysis.

### 2.4. In Vitro Assays

#### 2.4.1. RAW 264.7 Macrophage Culture and Pharmacological Treatments

RAW 264.7 cells were cultured and maintained in DMEM medium (Thermo Fisher Scientific, Waltham, Massachusetts, USA) containing 10% fetal bovine serum and 1% antibiotic solution—1000 U/ml penicillin G and 100 U/ml streptomycin sulfate (Thermo Fisher Scientific, Waltham, Massachusetts, USA). Cells (1 × 10^5^ cells/well) were treated with EAFPg (50–100 *μ*g/ml), kaempferol (25–100 *μ*g/ml), or dexamethasone (2–4 *μ*g/ml) and stimulated with LPS (10 *μ*g/ml; 30,000 EU/well/100 *μ*l; Sigma, St. Louis, MI, USA), at noncytotoxic concentrations. Then, cells were incubated for 48 h at 37°C and 5% CO_2_. Vehicle- (1% DMSO in PBS) treated cells were used as controls.

#### 2.4.2. RT-qPCR Assays in RAW 264.7 Murine Macrophages

TNF-*α*, IL-6, IL1-*β*, and IL-10 mRNA expression was evaluated in RAW 264.7 cells by RT-qPCR as described in Section 2.3.4.

#### 2.4.3. NO Levels in Cell Culture Supernatants

The production of NO was determined by measuring nitrite as previously described [7]. Briefly, cell supernatants were incubated with an equal volume of a Griess reagent (Sigma, St. Louis, MI, USA), and the absorbance was determined at 540 nm and compared with that obtained from a NO_2_ standard curve (0–300 *μ*M). Results are expressed as *μ*M of NO_2_.

### 2.5. Statistical Analysis

Data are expressed as mean ± standard deviation (SD). Differences between groups were analyzed by one-way analysis of variance (ANOVA), followed by the Newman-Keuls multiple comparison test. Percentages of inhibition were calculated as the mean of the inhibitions obtained for each individual experiment. *p* values < 0.05 were considered statistically significant.

## 3. Results

### 3.1. Phytochemical Analysis of EAFPg

**Figure 1:**
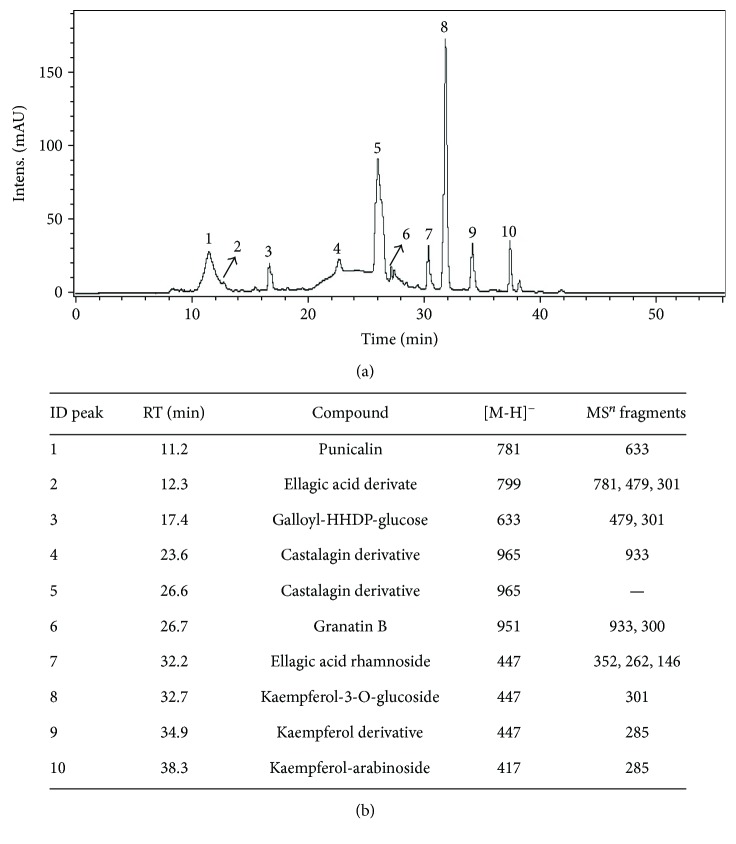
HPLC/DAD chromatogram of the acetyl acetate extract of pomegranate leaves (EAFPg) monitored at 327 nm (a). Structure of constituents identified by HPLC-DAD-ESI-IT/MS (b). ID: identification; RT: retention time; MS: mass spectrometer.

Figure 1 shows the HPLC analytical plot for EAFPg at 327 nm (a) and the detected components (b). These included punicalin (peak 1; retention time: 11.2 min), ellagic acid derivative (peak 2; retention time: 12.3 min), galloyl-HHDP-glucose (peak 3; retention time: 17.4 min), castalagin derivative (peaks 4 and 5; retention times: 23.6 and 26.6 min, resp.), granatin B (peak 6; retention time: 26.7 min), ellagic acid rhamnoside (peak 7; retention time: 32.2 min), kaempferol-3-O-glucoside (peak 8; retention time: 32.7 min), kaempferol derivative (peak 9; retention time: 34.9 min), and kaempferol-arabinoside (peak 10; retention time: 38.3 min).

### 3.2. EAFPg Reduces Leukocyte Accumulation and Collagen Deposition into the Lungs of Mice with ALI

**Figure 2:**
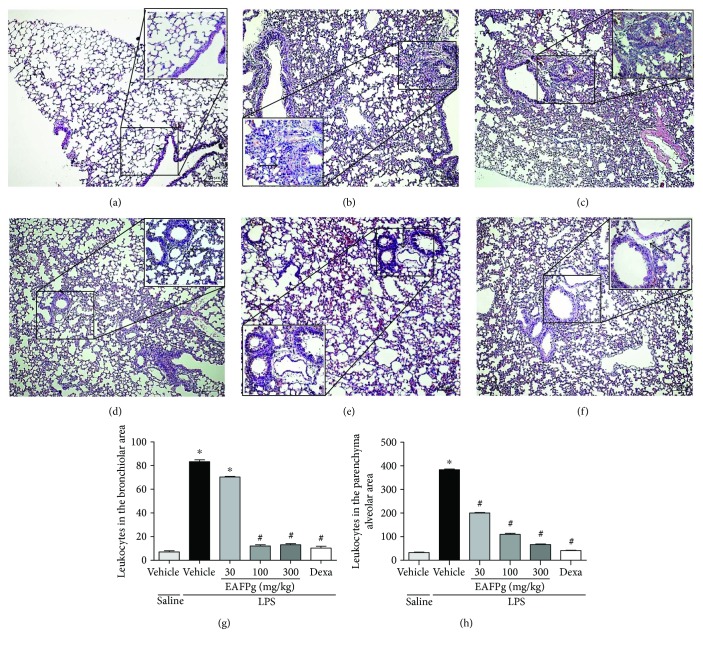
Effect of the pretreatment with EAFPg on leukocyte accumulation into the lungs obtained from mice treated with saline (a), LPS (b), 30 mg/kg EAFPg plus LPS (c), 100 mg/kg EAFPg plus LPS (d), 300 mg/kg EAFPg plus LPS (e), or 5 mg/kg dexamethasone plus LPS (f). Leukocyte counts in the bronchial (g) and alveolar parenchyma areas (h). Data is expressed as mean ± SD. Significances were calculated by one-way ANOVA followed by the Newman-Keuls multiple comparison test analysis. ^∗^*p* < 0.05 differs from saline-treated mice; ^#^*p* < 0.05 differs from vehicle-treated LPS-stimulated mice. Bronchiolar inflammatory cell infiltrates are shown as black arrows. Sections (4 *μ*m) were stained with hematoxylin and eosin. Magnification of 100x and 400x.

**Figure 3:**
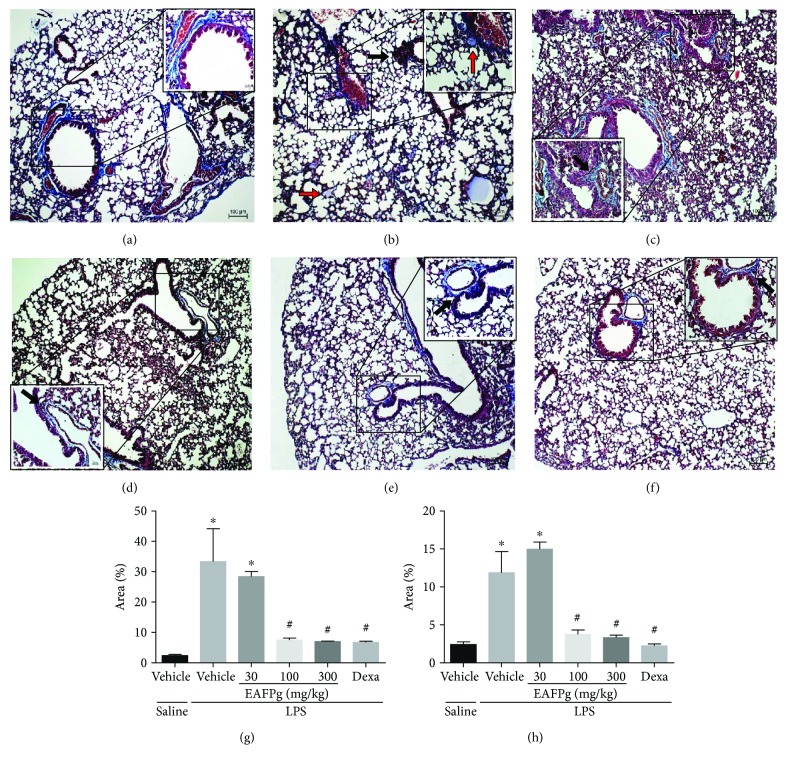
Effect of the pretreatment with EAFPg and dexamethasone on the collagen deposition in lung samples obtained from mice treated with saline (a), LPS (b), 30 mg/kg EAFPg plus LPS (c), 100 mg/kg EAFPg plus LPS (d), 300 mg/kg EAFPg plus LPS (e), or 5 mg/kg dexamethasone plus LPS (f). Quantitative analysis of the collagen area (percentage area) in the bronchial (g) and alveolar parenchyma areas (h). Data is expressed as mean ± SD. Significances were calculated by one-way ANOVA followed by the Newman-Keuls multiple comparison test analysis. ^∗^*p* < 0.05 differs from saline-treated mice; ^#^*p* < 0.05 differs from vehicle-treated LPS-stimulated mice. Collagen areas with leukocyte infiltration are shown as red arrows. Sections (4 *μ*m) were stained with Masson's trichrome. Magnification of 100x and 400x.

Figures 2 and 3 show the effects of EAFPg (30–300 mg/kg) and dexamethasone (5 mg/kg) on the lung tissue of mice with LPS-induced ALI. Animals with ALI exhibited an intense inflammatory cell influx surrounding the bronchi (Figure 2(b)). The same mice also presented multifocal areas of collagen deposition (Figure 3(b)) in comparison with those which received i.n. saline (Figure 3(a)). LPS-induced pathological changes were not affected by the pretreatment with EAFPg at 30 mg/kg. On the other hand, pretreatment with EAFPg at 100–300 mg/kg markedly reduced the collagen deposition with leukocyte infiltration, with respect to LPS mice treated with vehicle (Figures 2(d), 2(e), 3(d), and 3(e)). Animals pretreated with dexamethasone also displayed attenuated lung inflammation (Figures 2(f) and 3(f)). In addition, EAFPg (100–300 mg/kg) and dexamethasone markedly diminished the numbers of leukocytes in the bronchial area (12.0 ± 1.0, 13.0 ± 1.0, and 10.3 ± 1.5 leukocytes/130 *μ*m^2^, resp.) (Figure 2(g)) and in the alveolar parenchyma area (109.7 ± 4.7, 66.3 ± 2.5, and 41.3 ± 1.5 leukocytes/130 *μ*m^2^, resp.) when compared with those of the vehicle-treated ALI mice (83.3 ± 1.5 leukocytes/130 *μ*m^2^ and 384.3 ± 3.5 leukocytes/130 *μ*m^2^). Moreover, EAFPg (100–300 mg/kg) and dexamethasone markedly reduced the bronchial (7.5% ± 0.6%, 7.0% ± 0.1%, and 6.7% ± 0.3%, resp.; Figure 2(g)) and the alveolar parenchyma collagen deposition (3.7% ± 0.5%, 3.3% ± 0.7%, and 2.2% ± 0.2%, resp.) when compared with that of the ALI control group (33.3% ± 10.8% and 11.9% ± 2.7%, resp.).

### 3.3. EAFPg Reduces TNF-*α* and IL-1*β* but Not IL-6 mRNA Expression in the Lungs

**Figure 4:**
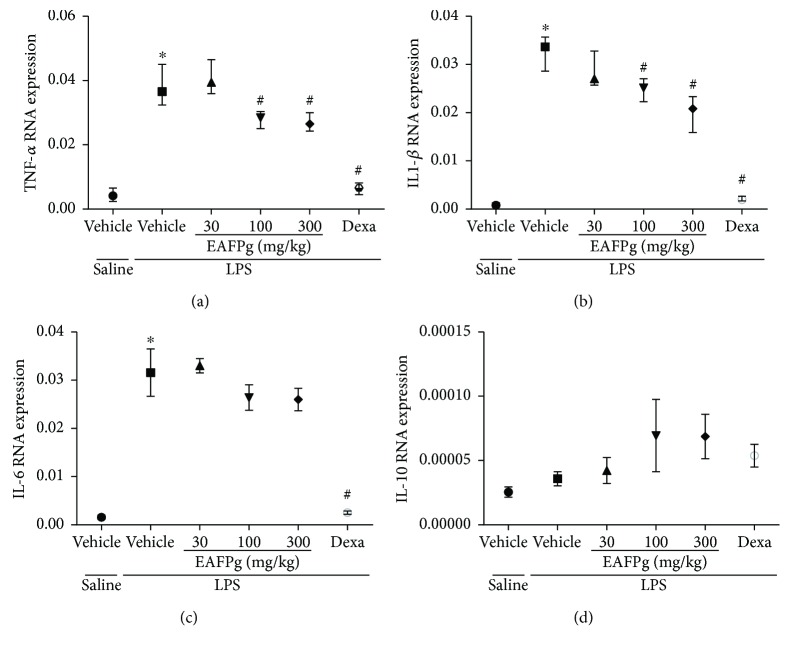
Effects of the pretreatment with EAFPg on TNF-*α* (a), IL-1*β* (b), IL-6 (c), and IL-10 gene expression in the lungs of the animals divided into six groups. Animals were randomly distributed into six groups (*n* = 6/group). Mice received in the prophylactic scheme EAFPg (30 mg/kg, 100 mg/kg, and 300 mg/kg, p.o.), dexamethasone (DEXA, 5 mg/kg, p.o.), or vehicle (PBS, p.o.). The values are presented as the median and interquartile range. Significances were calculated by one-way ANOVA followed by the Newman-Keuls multiple comparison test analysis. Groups versus nonpretreated saline-injected mice (^∗^*p* < 0.05) and versus nonpretreated LPS-installed mice (^#^*p* < 0.05).

LPS instillation significantly increased TNF-*α*, IL-1*β*, and IL-6 mRNA expression in the lungs (Figures 4(a)–4(c)). When tested at 30 mg/kg, EAFPg did not affect TNF-*α*, IL-1*β*, and IL-6 mRNA expression. On the other hand, 100 and 300 mg/kg EAFPG significantly downregulated lung TNF-*α* and IL-1*β* but not IL-6 expression in mice with ALI. Likewise, dexamethasone treatment significantly inhibited the expression of all evaluated cytokines, and the effects of the dexamethasone were significantly higher than those observed for EAFPg.

### 3.4. Plasma Extravasation and BALF TNF-*α* Levels Are Reduced in Mice with ALI Treated with EAFPg

**Figure 5:**
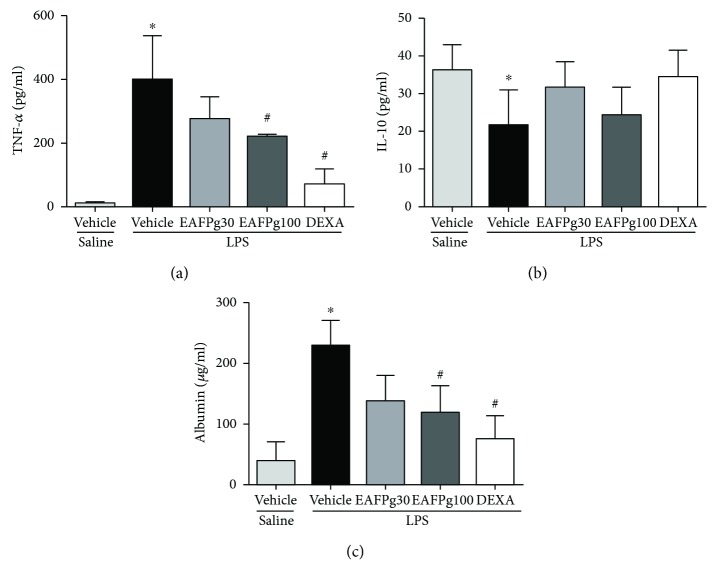
Effects of the pretreatment with EAFPg on TNF-*α* (a), IL-10 (b), and albumin (c) levels in BALF of the animals divided into six groups. Animals were randomly distributed into five groups (*n* = 6/group). Mice received in the prophylactic scheme EAFPg (30 mg/kg and 100 mg/kg, p.o.), dexamethasone (DEXA, 5 mg/kg, p.o.), or vehicle (PBS, p.o.). The values are presented as the mean ± SD. Significances were calculated by one-way ANOVA followed by the Newman-Keuls multiple comparison test analysis versus nonpretreated saline-injected mice (^∗^*p* < 0.05) and versus nonpretreated LPS-installed mice (^#^*p* < 0.05).

Albumin and TNF-*α* protein levels were augmented in BALF samples obtained from mice with ALI (Figures 5(a) and 5(c)) while IL-10 was reduced (Figure 5(b)). On the other hand, IL-10 was reduced in the BALF from the same animals. Both EAFPg (100 mg/kg) and dexamethasone diminished the levels of TNF-*α* (219.8 ± 6.4 pg/ml) and albumin (120.6 ± 42.8 pg/ml) in the BALF when compared with those of LPS-challenged mice pretreated with vehicle (397 ± 109.6 and 228.6 ± 41.6 pg/ml, resp.; Figures 5(a) and 5(c)). No effects were observed for either EAFPg or dexamethasone on IL-10 levels in the tested doses (Figure 5(b)).

### 3.5. EAFPg Prevents Leukocyte Accumulation into the Alveoli

**Figure 6:**
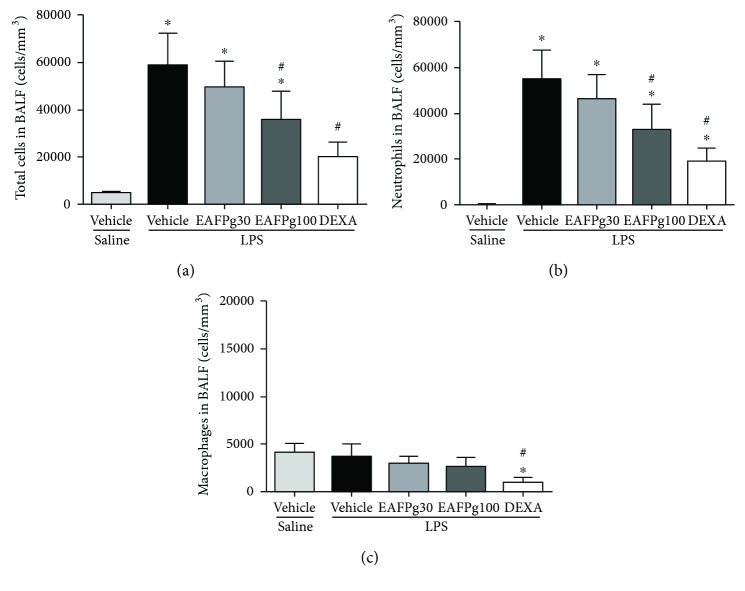
Effects of the pretreatment with EAFPg on the numbers of total leukocytes (a), neutrophils (b), or macrophages (c) in BALF and total leukocytes in the lungs. Animals were randomly distributed into five groups (*n* = 6/group). Mice received in the prophylactic scheme EAFPg (30 mg/kg and 100 mg/kg, p.o.), dexamethasone (DEXA, 10 mg/kg, p.o.), or vehicle (PBS, p.o.). The values are presented as the mean ± SD. Significances were calculated by one-way ANOVA followed by the Newman-Keuls multiple comparison test analysis versus nonpretreated saline-injected mice (^∗^*p* < 0.05) and versus nonpretreated LPS-installed mice (^#^*p* < 0.05).

Mice with LPS-induced ALI had increased numbers of total and differential leukocytes in their BALF in comparison with the saline group (Figures 6(a), 6(b), and 6(c)). In contrast, EAFPg- (100 mg/kg) pretreated mice with ALI had significantly lower numbers of leukocytes (35,814 ± 12,005 cells/mm^3^) in their BALF in comparison with vehicle-treated mice (58,733 ± 13,629 cells/mm^3^, *p* < 0.0001, see Figure 6(a)). A similar effect was noted for LPS-challenged mice pretreated with dexamethasone (Figure 6(a)). In addition, the number of neutrophils was significantly reduced in LPS-challenged mice pretreated with 100 mg/kg of EAFPg (32,878 ± 11,116 cells/mm^3^) when compared with vehicle-treated mice (54,813 ± 12,678 cells/mm^3^, *p* < 0.0001, see Figure 6(b)). No differences were observed in the number of macrophages between groups (Figure 6(c)).

### 3.6. EAFPg, Kaempferol, and Dexamethasone Reduce Cytokine mRNA Expression in Cultured RAW 264.7 Macrophages

**Figure 7:**
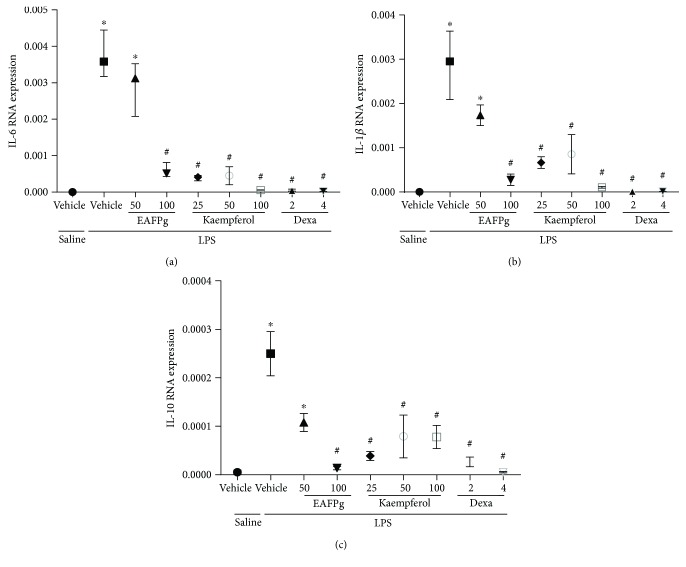
Effect of the pretreatment with EAFPg (50–100 *μ*g/ml) or kaempferol (25–100 *μ*g/ml) on IL-6 (a), IL-1*β* (b), and IL-10 (c) mRNA expression in RAW 264.7 macrophages. Data is expressed as mean ± SD. Significances were calculated by one-way ANOVA followed by the Newman-Keuls multiple comparison test analysis. ^∗^*p* < 0.05 differs from saline-treated cells; ^#^*p* < 0.05 differs from vehicle-treated LPS-stimulated cells.

IL-1*β*, IL-6, and IL-10 mRNA levels were downregulated by EAFPg (100 *μ*g/ml), kaempferol (25–100 *μ*g/ml), and dexamethasone (2–4 *μ*g/ml) in macrophages activated by LPS (Figures 7(a), 7(b), and 7(c)).

### 3.7. EAFPg and Kaempferol Reduce NO Release by Macrophages

**Figure 8:**
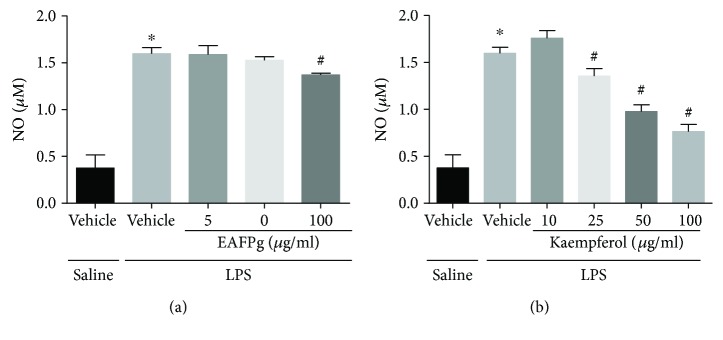
Effect of the pretreatment with EAFPg (50–100 *μ*g/ml) (a) or kaempferol (25–100 *μ*g/ml) (b) on NO release by RAW 264.7 macrophages. Data is expressed as mean ± SD. Significances were calculated by one-way ANOVA followed by the Newman-Keuls multiple comparison test analysis. ^∗^*p* < 0.05 differs from saline-treated cells; ^#^*p* < 0.05 differs from vehicle-treated LPS-stimulated cells.

NO production was reduced in LPS-activated macrophages incubated with EAFPg (100 *μ*g/ml) and kaempferol (25–100 *μ*g/ml) (Figures 8(a) and 8(b), resp.).

## 4. Discussion

Extracts prepared from pomegranate peels and seeds were described previously to possess anti-inflammatory properties [12, 13]. However, very little is known about the effects of pomegranate leaf extracts or fractions on inflammation. We recently demonstrated that the pomegranate leaf hydroalcoholic extract attenuates LPS-induced peritonitis by decreasing the accumulation of leukocytes and the release of proinflammatory mediators in the peritoneum [8]. We now show that EAFPg protects mice from ALI by reducing lung inflammation.

The phytochemical analysis of EAFPg demonstrated the presence of kaempferol, ellagic acid derivative, and other bioactive molecules, a similar composition to that previously observed for the leaf hydroalcoholic extract [8]. Chen et al. [14] showed that kaempferol significantly blocks LPS-induced activation of mitogen-activated protein kinases (MAPKs) and nuclear factor-kappa B (NF-*κ*B). In addition, Cornelio Favarin et al. demonstrated that ellagic acid reduces cyclooxygenase-2- (COX-2-) induced exacerbation of inflammation, vascular permeability changes, and neutrophil recruitment to the BALF of mice with ALI induced by acid [15]. However, kaempferol was found to be the major compound in EAFPg. Thus, we hypothesized that EAFPg is anti-inflammatory probably due to the content of kaempferol in its composition.

In patients with ALI, one of the main pathological changes observed in their lungs is an intense inflammatory infiltrate as a consequence of the increased permeability of the alveolar-capillary barrier and epithelial damage. In our study, it was observed that EAFPg suppresses lung inflammation and collagen deposition in the airways of mice intranasally challenged with LPS. Corroborating these findings, de Oliveira et al. [10] showed that the crude extract obtained from pomegranate leaves inhibits the pathological changes induced by ovalbumin in the lung tissue.

The inflammatory infiltrate observed in ALI results from intense and persistent inflammation. Initially, the inflammation is provoked by lung-resident macrophages that activate intracellular mediators after interacting with pathogen-associated molecular patterns, such as LPS. It was not observed that increased migration of monocyte-derived macrophages to the lung suggested that 4 hours is not enough for its migration. Large amounts of cytokines were primarily produced and released by lung-resident macrophages in response to LPS [16]. Therefore, to further evaluate the effects of the EAFPg on lung inflammation, we measured the inflammatory markers TNF-*α*, IL1-*β*, and IL-6. We found that the pretreatment with EAFPg reduces the gene expression of these mediators in the lung tissue. TNF-*α* protein levels were also attenuated in the same mice. Additionally, cytokine mRNA expression was potently attenuated in LPS-stimulated macrophages treated with EAFPg or kaempferol. It was previously demonstrated that the hydroalcoholic extract from pomegranate leaves reduces TNF-*α* mRNA expression in peritoneal leukocytes obtained from rats with LPS-induced peritonitis [8].

TNF-*α* induces the expression of adhesion molecules on endothelial cells, thus contributing to neutrophil migration. In ALI, neutrophils are the first cells to be recruited into the lungs [17]. In the present study, we demonstrated that EAFPg reduces neutrophil migration into the alveoli. Accordingly, Bachoual et al. [18] showed that an aqueous extract of *P. granatum* peel inhibits neutrophil-mediated myeloperoxidase activity and attenuates LPS-induced lung inflammation in mice. In addition, Marques et al. [8] showed that the numbers of peritoneal leukocytes, especially neutrophils, are reduced in hydroalcoholic extract-treated rats with acute peritonitis.

Taken together, our data demonstrate that EAFPg may be used as food supplements, like tea, to prevent inflammatory disorders in which neutrophils play an essential role, such as ALI. However, further studies are necessary in order to address the effects of EAFPg in later time points of ALI.
